# Comparing Bacterial Community Composition between Healthy and White Plague-Like Disease States in *Orbicella annularis* Using PhyloChip™ G3 Microarrays

**DOI:** 10.1371/journal.pone.0079801

**Published:** 2013-11-20

**Authors:** Christina A. Kellogg, Yvette M. Piceno, Lauren M. Tom, Todd Z. DeSantis, Michael A. Gray, David G. Zawada, Gary L. Andersen

**Affiliations:** 1 United States Geological Survey, St. Petersburg Coastal and Marine Science Center, St. Petersburg, Florida, United States of America; 2 Lawrence Berkeley National Laboratory, Berkeley, California, United States of America; 3 Second Genome, Inc., San Bruno, California, United States of America; Pennsylvania State University, United States of America

## Abstract

Coral disease is a global problem. Diseases are typically named or described based on macroscopic changes, but broad signs of coral distress such as tissue loss or discoloration are unlikely to be specific to a particular pathogen. For example, there appear to be multiple diseases that manifest the rapid tissue loss that characterizes ‘white plague.’ PhyloChip™ G3 microarrays were used to compare the bacterial community composition of both healthy and white plague-like diseased corals. Samples of lobed star coral (*Orbicella annularis*, formerly of the genus *Montastraea* [1]) were collected from two geographically distinct areas, Dry Tortugas National Park and Virgin Islands National Park, to determine if there were biogeographic differences between the diseases. In fact, all diseased samples clustered together, however there was no consistent link to *Aurantimonas coralicida*, which has been described as the causative agent of white plague type II. The microarrays revealed a large amount of bacterial heterogeneity within the healthy corals and less diversity in the diseased corals. Gram-positive bacterial groups (Actinobacteria, Firmicutes) comprised a greater proportion of the operational taxonomic units (OTUs) unique to healthy samples. Diseased samples were enriched in OTUs from the families Corynebacteriaceae, Lachnospiraceae, Rhodobacteraceae, and Streptococcaceae. Much previous coral disease work has used clone libraries, which seem to be methodologically biased toward recovery of Gram-negative bacterial sequences and may therefore have missed the importance of Gram-positive groups. The PhyloChip™data presented here provide a broader characterization of the bacterial community changes that occur within *Orbicella annularis* during the shift from a healthy to diseased state.

## Introduction

Coral disease is now recognized as one of the major causes of reef degradation and coral mortality. The Caribbean has been particularly hard hit, with disease mortality reducing coral cover by an order of magnitude in some areas [2], [3]. A frustrating aspect of trying to manage or prevent disease is that corals have a limited number of ways to visually manifest distress (e.g., discoloration, tissue loss) and as such, the macroscopic signs that ‘define’ a disease actually have minimal diagnostic value. Using tissue loss to characterize a coral disease is on the order of characterizing a human disease solely by the presence of a fever. Being unable to clearly distinguish one disease from another, or link macroscopic signs to specific microbial pathogens, makes it difficult, if not impossible, to develop a plan for containment or prevention.

White plague is a perfect example. This disease is visibly ‘identified’ by a sharp boundary between pigmented tissue and bare skeleton that is bright white due to recent tissue loss [4], [5]. In the Caribbean, three distinct ‘types’ (WPI, WPII, WPIII) have been described, differentiated only by the progression speed of the lesion [4], [6]. One problem is that most microbial studies have collected samples at a single time point without any information about the progression speed. Therefore, each could be describing a different ‘type,’ while combining the microbial results under the single disease name. To further underscore the point that this macroscopic symptom is not diagnostic enough, Red Sea corals that appeared to have white plague were found by microbiology and histology to be suffering from an atypical black band disease [7]. Even when confined to Atlantic corals, there appear to be numerous white plague-like diseases [8]–[13]. Fortunately, researchers are beginning to realize the confusion this has created and it is becoming more common to either distinguish a disease as ‘white plague-like’ if no progression data exists, or give each outbreak a more descriptive name (e.g., “Tortugas multispecies rapid tissue loss disease” [8]) following the recommendations offered by Work and Aeby [14].

Although causative bacterial pathogens have been identified for two white plague diseases (*Aurantimonas coralicida*, for WPII in the Caribbean [15] and *Thalassomonas loyana* for white plague-like disease in the Red Sea [16]), it is increasingly believed that coral diseases arise from environmental stress triggering opportunistic pathogens rather than from primary pathogens [17], [18]. Therefore, the causative agent of a disease or syndrome may not always be the same, especially in different geographic areas. Many previous studies of coral disease have been culture-based [10], [19], [20], [21] or relied on clone libraries of a few hundred sequences [12], [22], [23], [24], both of which give a limited characterization of the microbial community diversity. Within the last five years, there has been a shift towards techniques that provide a more comprehensive view of the coral-associated microbial community in healthy and diseased corals, such as PhyloChip™ DNA microarrays [25], [26] and pyrosequencing [27]–[29]. The objective of this work was to use PhyloChip™ G3 microarrays to examine changes in the coral-associated bacterial communities between healthy *Orbicella annularis* (formerly of the genus *Montastraea* [1]) coral colonies and those affected by white-plague-like disease. The PhyloChip™ G3 (generation 3) microarray [30] provides a broad taxonomic overview of microbial communities. This microarray is printed with 1.1 million 16S rRNA gene probes representing nearly 60,000 operational taxonomic units (OTUs) that fit hierarchically into taxa from domain to species. This extensive coverage of known bacterial taxa allows detection of more bacterial richness than traditional clone library analysis. The large but standardized OTU inventory allows for easier analysis than an open-ended pyrosequencing dataset. Samples were compared from the Dry Tortugas and Virgin Islands to test whether the disease patterns clustered based on geographic areas, to look for diagnostic taxonomic shifts between healthy and diseased samples, and to see if either location’s disease was associated with *Aurantimonas coralicida*, the causative agent identified for White Plague Type II.

## Materials and Methods

### Ethics Statement

These collections were made under permits VIIS-2008-SCI-0033 (study VIIS-08033) and DRTO-2009-SCI-0018 (study DRTO-00074), granted to the first author by the Virgin Islands National Park and Dry Tortugas National Park, respectively. No ethical approval was required for the experimental research described here.

### Sample Sites and Collections

Healthy and white plague-affected *Orbicella annularis* (lobed star coral) samples were collected in two National Parks during the summer of 2009. Five healthy and six diseased colonies were sampled at Tektite Reef in Great Lameshur Bay (18°18′46 N, 64°43′22 W), St. John, Virgin Islands National Park (VIIS) on July 16–17 (Table 1). Samples from five healthy and three diseased colonies were also collected from the ‘Little Africa’ patch reef (24°38′06 N, 82°55′15 W) in the Dry Tortugas National Park (DRTO) on August 5 (Table 1). Water temperature was 31–32°C with a salinity of 35 ppt at DRTO and 29°C with a salinity of 34 ppt at VIIS. Healthy corals were normally pigmented with no lesions or tissue loss. White plague-affected corals exhibited a sharp interface between normal tissue and freshly exposed skeleton.

**Table 1:** *Orbicella annularis* samples collected for microarray analysis.

Sample ID	Location	Date Collected	Health State	Depth (m)
VIISMAP01	Virgin Islands National Park	July 16, 2009	white plague	12
VIISMAP02	Virgin Islands National Park	July 16, 2009	white plague	12
VIISMAP03	Virgin Islands National Park	July 16, 2009	white plague	13
VIISMAP04	Virgin Islands National Park	July 17, 2009	white plague	8
VIISMAP05	Virgin Islands National Park	July 17, 2009	white plague	6
VIISMAH06	Virgin Islands National Park	July 17, 2009	healthy	9
VIISMAH07	Virgin Islands National Park	July 17, 2009	healthy	8
VIISMAH09	Virgin Islands National Park	July 17, 2009	healthy	5
VIISMAH10	Virgin Islands National Park	July 17, 2009	healthy	6
VIISMAP11	Virgin Islands National Park	July 17, 2009	white plague	7
DRTOMAP01	Dry Tortugas National Park	Aug 5, 2009	white plague	3
DRTOMAP02	Dry Tortugas National Park	Aug 5, 2009	white plague	2
DRTOMAP03	Dry Tortugas National Park	Aug 5, 2009	white plague	2
DRTOMAH05	Dry Tortugas National Park	Aug 5, 2009	healthy	2
DRTOMAH06	Dry Tortugas National Park	Aug 5, 2009	healthy	3
DRTOMAH07	Dry Tortugas National Park	Aug 5, 2009	healthy	3
DRTOMAH08	Dry Tortugas National Park	Aug 5, 2009	healthy	3
DRTOMAH09	Dry Tortugas National Park	Aug 5, 2009	healthy	3

A healthy sample designated VIISMAH08 was collected but no 16S rRNA gene PCR product was obtained, so it was not included in this table since it does not appear in any subsequent figures.

Note that in spite of extensive searching, the three samples of white plague-like disease collected in DRTO were the only ones encountered.

Corals were sampled using a bleach-sterilized metal punch of approximately 2-cm diameter to collect two biopsies of tissue with minimal skeletal material attached. Diseased corals were sampled at the lesion interface. Coral tissue samples were immediately placed into sterile Whirl-pak bags. The punch was cleaned and re-bleached between samples to prevent any transfer of microbes or contamination. Back on shore, the coral samples were briefly rinsed with sterile-filtered seawater to remove loosely associated microbes, wrapped in sterile aluminum foil, placed into a fresh Whirl-pak bag, and flash-frozen in liquid nitrogen. Samples were transported from the field to the USGS St. Petersburg Science Center and then transferred to a −80°C freezer for storage.

### DNA Extraction

In the laboratory, each pair of frozen tissue samples from a single coral colony was ground to powder using a sterile mortar and pestle on dry ice and then combined. The microbial community DNA was extracted in duplicate from each combined sample using the MO BIO PowerPlant® DNA Isolation Kit with some modification as previously described by Sunagawa et al. [31]. At the end of the protocol, the replicate extractions were combined, resulting in a single microbial DNA extraction representing each coral colony sampled.

### 16S rRNA Gene Amplification

Amplification was carried out as previously described [32]. Primers targeting 16S bacterial ribosomal RNA genes were used: 27F with the degenerate base removed, sometimes also referred to as 8F (5′-AGAGTTTGATCCTGGCTCAG-3′) [33] and 1492R (5′-GGTTACCTTGTTACGACTT-3′) [34]. Eight replicate 25 µl amplifications were performed for each DNA extraction over a temperature gradient of 48–58°C. At each temperature, 2 µl of template DNA was used. Final concentrations for each 25 µl reaction were: 1× Ex Taq Buffer with 2 mM MgCl^2^, 300 nM each primer (27F and 1492R), 200 µM each dNTP, 25 µg bovine serum albumin and 0.625 units of Ex Taq polymerase. The reactions were amplified using an iCycler and under the following thermocycling conditions: 95°C for 3 min for initial denaturation, 30 cycles of 95°C for 30 sec, 48–58°C for 30 sec, and 72°C for 2 min, and then final extension for 10 min at 72°C. PCR products from each annealing temperature for a sample were combined and concentrated using Amicon Ultra-0.5, Ultracel-30 Membrane, 30 kDa centrifugal filter units and quantified before application to the PhyloChip™. The concentrated 1 µl PCR product was quantified on a 2% agarose E-gel using the Low Range Quantitative DNA Ladder as reference. In some cases the primers amplified both coral and bacterial DNA [35], requiring gel extraction of the bacterial amplicons for all samples to be consistent. The difference in size between the two products (i.e., coral and bacterial amplicons) was roughly 200 base pairs. To allow clear separation of the target (bacterial) amplicon, gels were run twice as long as usual before being stopped to excise the 16S rRNA amplicon.

### PhyloChip™ G3 Analysis

Details about probe selection, probe scoring, data acquisition and preliminary data analysis are according to Hazen et al. [30]. For each sample, 500 ng of PCR product was applied to an individual PhyloChip™ G3 DNA microarray, with the exception of one microarray (representing sample VIISMAH06), which received 450 ng because of less robust amplification. To summarize the procedure, the sample 16S rRNA PCR products and a mixture of PCR products at known concentrations (referred to as ‘spike-mix’) were combined, fragmented using DNAseI, and biotin-labeled using the recommended protocol for Affymetrix Prokaryotic Arrays. These labeled products were hybridized on the PhyloChip™ G3 overnight at 48°C while rotating at 60 rpm. The microarrays were washed, stained, and scanned as previously described [30]. Briefly: Fluorescent images were capture with the GeneChip Scanner 3000 7G. Each 25mer probe array feature occupied approximately 8×8 pixels in the image file. The central 9 pixels were ranked by intensity, and the 75^th^ percentile was used as the summary intensity for the feature. Array fluorescence intensities could range from 1 to 65,536. The hybridization score (HybScore, a.k.a., OTU intensity) for an OTU was calculated as the mean intensity of the perfectly matching probes exclusive of the maximum and minimum. HybScore data obtained from the CEL files (produced from GeneChip Microarray Analysis Suite, version 5.1) were scaled by setting the spike-mix control intensity to 10,000 to correct for slight differences in scan intensities or potential uneven hybridization efficiencies across microarrays.

### Data Analyses

An updated taxonomy [36] was used for this dataset (updated since [32]). Stage 1 of PhyCA analysis was performed to remove OTUs not present in any of the samples using cutoff values: q1 = 0.5, q2 = 0.93, q3 = 0.98). This reduced the total number of OTUs subject to analysis from roughly 60,000 down to 13,441 (Table 2). The microarray data has been archived online at Greengenes, accessible by the following dedicated URL: (http://greengenes.lbl.gov/Download/Microarray_Data/Orbicella_Kellogg/). Intensity values were log_2_ transformed before hybridization data were further analyzed.

**Table 2:** Enumeration of taxonomic rank members detected across replicate PhyloChips™ from healthy and diseased coral samples (after Sunagawa et al, 2009).

All OTUs								
TaxonomicRank	Healthy(abs.)	Diseased(abs.)	PercentDecrease	UniqueHealthy (%)	UniqueDiseased (%)		Shared Healthy &Diseased (%)	Total
OTUs	11,869	6,346	46.5	7,095 (52.8)	1,572 (11.7)		4,774 (35.5)	13,441
Species	627	388	38.0	307 (44.2)	67 (9.6)		321 (46.2)	695
Genera	775	569	26.6	246 (30.2)	40 (4.9)		529 (64.9)	815
Families	333	280	15.9	61 (17.9)	8 (2.3)		272 (79.8)	341
Orders	185	165	10.8	25 (13.2)	5 (2.6)		160 (84.2)	190
Classes	126	109	13.5	23 (17.4)	6 (4.6)		103 (78.0)	132
Phyla	73	61	16.4	12 (16.4)	0 (0)		61 (83.6)	73
**Singletons Removed**								
**Taxonomic** **Rank**	**Healthy** **(abs.)**	**Diseased** **(abs.)**	**Percent** **Decrease**	**Unique** **Healthy (%)**	**Unique** **Diseased (%)**	**Shared Healthy &** **Diseased (%)**		**Total**
OTUs	4,599	3,555	28.0	1,519 (22.6)	428 (6.4)	4,774 (71.0)		6,721
Species	281	229	18.5	66 (17.0)	20 (5.1)	303 (77.9)		389
Genera	470	407	13.4	33 (6.0)	18 (3.3)	497 (90.7)		548
Families	257	230	10.5	19 (6.7)	2 (0.7)	263 (92.6)		284
Orders	152	142	6.6	11 (6.5)	2 (1.2)	155 (92.3)		168
Classes	94	94	0	2 (1.9)	2 (1.9)	101 (96.2)		105
Phyla	59	54	8.5	5 (7.7)	1 (1.5)	59 (90.8)		65

Top panel includes all OTUs. Bottom panel excludes singletons (defined as OTUs present only in a single sample).

**Figure 1:**
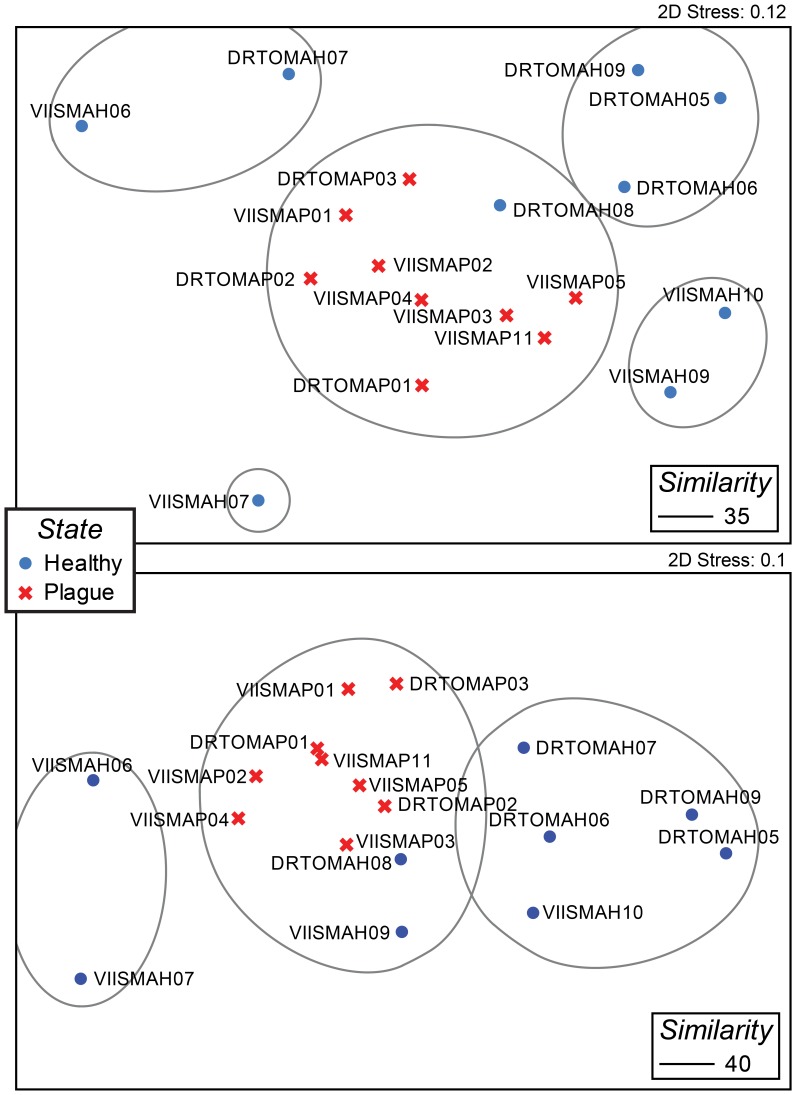
Non-metric multidimensional scaling (NMDS) plot of samples, based on Bray-Curtis similarity of presence/absence OTU data. Panel A is based on total OTUs (13,441). Panel B is based on the subset of OTUs that include at least one coral-associated sequence (313).

PRIMER 6 version 6.1.13 software [37] was used to generate cluster analyses and nonmetric multidimensional scaling (NMDS) plots to examine sample grouping (Figure 1). When using binary presence/absence data, no transformation was performed prior to computing the Bray Curtis similarities. Square-root transformed intensity data were used to calculate the two-way crossed analyses of similarity (ANOSIM) to test for significant differences in the bacterial community composition between predefined sample sets (i.e., based on collection location and health state).

A custom PERL script was created (QueryOTU.pl, File S1) to screen the results for unique OTUs. The input file consisted of a modified OTU table (File S2) of binary presence/absence data with one extra column added labeled “Outgroup” which had zeros in all the real OTU rows and one arbitrary OTU numbered 999999. Running the PERL script on the.csv file generates a sequentially numbered list of the samples, followed by a prompts to choose the samples to include in Group A and then Group B. The program then compares the two groups, excluding any OTUs found in both, to provide a list of OTUs unique to each. The output includes the total number of OTUs per group, a list of the OTU numbers, and which samples within the group contain each OTU. The outgroup OTU and sample were included to allow determinations of total OTUs including those shared by other real samples. For a more detailed description of how this program was used to obtain the data in Table 2, please see File S3.

A QIIME-formatted OTU table based on the presence/absence data and a mapping file were created and run through QIIME [38]. The script ‘summarize_taxa_through_plots.py’ was used to generate taxa summary plots of OTU abundance at the phylum through family levels. This script was also used to compare grouped healthy samples versus grouped diseased samples by passing the –c option.

## Results

There was a significant difference between healthy and diseased samples (Figure 1A; ANOSIM Global R = 0.316, p = 0.025) and the Global R value supports moderately distinct microbial communities in the diseased and healthy coral tissues. Although the depth range of collections was from 2 to 13 meters, there does not appear to be any structuring based on depth (Table 1, Figure 1). Healthy samples showed a greater taxonomic diversity compared to diseased samples (Table 2). Out of the 7,095 OTUs unique to healthy samples, 106 (0.01%) were present in greater than 50% of the healthy replicates (Table 3). The majority (94/106) consist of Gram-positive bacteria from the phyla Actinobacteria and Firmicutes.

**Table 3:** Operational taxonomic units (OTUs) unique to healthy samples and present in greater than 50% (five or more) healthy replicates.

OTUs	Phylum	Class	Order	Family	Genus
1	Actinobacteria	Actinobacteria	Actinomycetales	Microbacteriaceae	*Cryobacterium*
1	Actinobacteria	Actinobacteria	Actinomycetales	Micrococcaceae	*Arthrobacter*
8	Actinobacteria	Actinobacteria	Actinomycetales	Micrococcaceae	*Micrococcus*
1	Actinobacteria	Actinobacteria	Actinomycetales	Propionibacteriaceae	*Propionibacterium*
1	Bacteroidetes	Flavobacteria	Flavobacteriales	Flavobacteriaceae	unclassified
1	Bacteroidetes	Sphingobacteria	Sphingobacteriales	Flammeovirgaceae	Candidatus Cardinium
81	Firmicutes	Bacilli	Bacillales	Bacillaceae	*Bacillus*
1	Firmicutes	Clostridia	Clostridiales	Clostridiaceae	*Clostridium*
1	Firmicutes	Clostridia	Clostridiales	Lachnospiraceae	*Coprococcus*
9	Proteobacteria	Gammaproteobacteria	Vibrionales	Vibrionaceae	*Vibrio*
1	Verrucomicrobia	Spartobacteria	Spartobacteriales	Spartobacteriaceae	MC18
**106**					

One of the major upgrades of the PhyloChip™ G3 over the previous generation (G2) is the representation of many coral-associated sequences in the additional OTUs. Through keyword searches followed by manual screening of the results, we determined that there are 545 total OTUs on the PhyloChip™ G3 that include at least one coral-associated sequence (Table S1). The bulk of these coral-associated sequences come from five studies [23], [26], [31], [39], [40]. Our *Orbicella annularis* dataset included 313 of these coral-associated OTUs. Out of the 1,572 OTUs unique to diseased samples (Table 2), only 38 (0.02%) were present in greater than 50% of the replicates (Table 4). These were pretty evenly split between Gram-positive and Gram-negative bacteria, with the main phyla being Firmicutes and Proteobacteria. Eighteen percent (7/38) of those OTUs represent sequences previously associated with corals (Table S1).

**Table 4:** Operational taxonomic units (OTUs) unique to diseased samples and present in ≥50% (five or more) diseased replicates.

OTUs	Phylum	Class	Order	Family	Genus
5	Actinobacteria	Actinobacteria	Actinomycetales	Corynebacteriaceae	*Corynebacterium*
1	Chloroflexi	Anaerolineae	Anaerolineales	Anaerolinaceae	A4b
1	Cyanobacteria	Chloroplast	Chlorophyta	Ulvophyceae	unclassified
1	Cyanobacteria	Oscillatoriophycideae	Chroococcales	Microcystaceae	*Microcystis*
1	Firmicutes	Bacilli	Bacillales	Staphylococcaceae	*Staphylococcus*
1	Firmicutes	Bacilli	Lactobacillales	Carnobacteriaceae	unclassified
1	Firmicutes	Bacilli	Lactobacillales	Lactobacillaceae	*Lactobacillus*
3	Firmicutes	Bacilli	Lactobacillales	Streptococcaceae	*Streptococcus*
2	Firmicutes	Clostridia	Clostridiales	Clostridiaceae	*Clostridium*
1	Firmicutes	Clostridia	Clostridiales	Incertae Sedis	*Sedimentibacter*
1	Firmicutes	Clostridia	Clostridiales	Lachnospiraceae	*Blautia*
2	Firmicutes	Clostridia	Clostridiales	Lachnospiraceae	*Roseburia*
2	Firmicutes	Clostridia	Clostridiales	Lachnospiraceae	unclassified
1	Firmicutes	Clostridia	Clostridiales	Ruminococcaceae	*Faecalibacterium*
1	Proteobacteria	Alphaproteobacteria	Rhizobiales	Cohaesibacteraceae	*Cohaesibacter*
1	Proteobacteria	Alphaproteobacteria	Rhodobacterales	Rhodobacteraceae	*Loktanella*
1	Proteobacteria	Alphaproteobacteria	Rhodobacterales	Rhodobacteraceae	*Paracoccus*
1	Proteobacteria	Alphaproteobacteria	Rhodobacterales	Rhodobacteraceae	*Rhodovulum*
1	Proteobacteria	Alphaproteobacteria	Rhodobacterales	Rhodobacteraceae	*Shimia*
1	Proteobacteria	Alphaproteobacteria	Rhodobacterales	Rhodobacteraceae	*Thalassobius*
3	Proteobacteria	Alphaproteobacteria	Rhodobacterales	Rhodobacteraceae	unclassified
1	Proteobacteria	Alphaproteobacteria	Rhodospirillales	Acetobacteraceae	*Asaia*
1	Proteobacteria	Alphaproteobacteria	Sphingomonadales	Sphingomonadaceae	*Novosphingobium*
1	Proteobacteria	Betaproteobacteria	Burkholderiales	Aquabacteriaceae	*Aquabacterium*
1	Proteobacteria	Deltaproteobacteria	Desulfovibrionales	Desulfovibrionaceae	*Desulfovibrio*
1	Proteobacteria	Deltaproteobacteria	Desulfuromonadales	Desulfuromonadaceae	*Desulfuromonas*
1	Proteobacteria	Gammaproteobacteria	Oceanospirillales	Alteromonadaceae	nsmpVI18
**38**					

Given such high representation of coral-associated sequences in the subset of OTUs unique to diseased samples, we reran the NMDS plot using only the 313 coral-associated OTUs that had been identified within the total dataset of 13,441 OTUs (Figure 1B). The consistency of the disease samples clustering together (compare Figure 1 panels A and B) indicates that these coral-associated sequences are important in distinguishing the health state of the samples.

**Figure 2:**
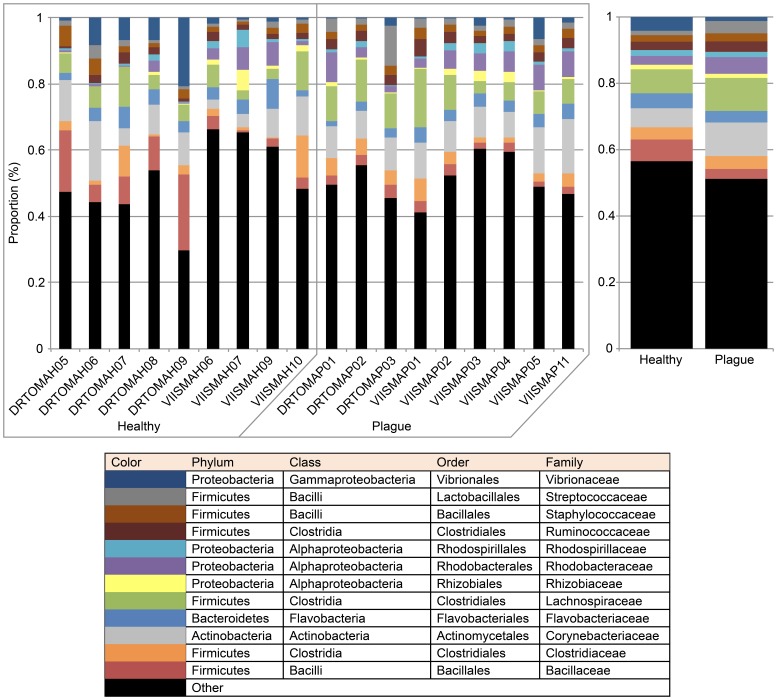
Relative diversity within bacterial families associated with healthy and white plague-like diseased corals. The 12 families shown were those representing greater than 5% of at least one sample. The remaining families are collectively represented by the ‘other’ category. The size of each color block (assigned to families in the key below) represents the number of OTUs detected in the family relative to the total number of OTUs detected in that sample. Individual samples are presented on the left and cumulative healthy and diseased profiles on the right.

A large number of taxa are shared by healthy and diseased samples at all phylogenetic levels (Table 2), but some patterns can be seen at the family level (Figure 2). When all healthy and plague samples are combined such that the most common families can be compared, there are differences in several groups (Figure 2). There are more Vibrionaceae and Bacillaceae OTUs in healthy samples than diseased samples, which correlate well with the presence of a number of healthy-specific OTUs from those families (Table 3). Similarly, the families Corynebacteriaceae, Lachnospiraceae, Rhodobacteraceae, and Streptococcaceae are better represented in diseased samples, echoing the presence of disease-specific OTUs (Table 4).

When comparing entire bacterial communities, the white plague-like disease samples all clustered together and there was no significant difference between the two sampling locations (ANOSIM Global R = 0.111, p = 0.187; Figure 1). This clustering indicates that the diseased samples have similar bacterial community patterns, regardless of geography.

With the PhyloChip™ G3 microarray, the presence of each OTU is reported based on the responses of multiple probes assigned to the OTU. An OTU may encompass a single unique sequence or several hundred from a reference database. The OTU that includes *Aurantimonas coralicida* WP1 (the causative agent for WPII; [15]) is not exclusive to that sequence, and so a positive result, while suggesting the presence of this bacterium, could nonetheless indicate the presence of another related bacterium. Only three samples were positive for this OTU at the cutoff values used: VIISMAP04, VIISMAH06 and VIISMAH07. All three samples were from the Virgin Islands, from one diseased colony and two visibly healthy colonies.

## Discussion

Using presence/absence of OTUs in our PhyloChip™results, we constructed non-metric multidimensional scaling plots that showed strong clustering of all the white plague-like samples, regardless of collection site (Figure 1). A PhyloChip™ G3 study that examined two coral species affected by a white plague-like disease on a single reef in the Indo-Pacific found similarity in the disease community irrespective of host [25]. These patterns of similar bacterial communities associated with disease states that are conserved across hosts [25] or locations (this study), suggest that the PhyloChip™ G3 may be a useful tool to differentiate and track specific coral diseases. This is in contrast to a study that examined *Orbicella annularis* from the Bahamas using length heterogeneity PCR (LH-PCR) to generate the OTUs [10]. In that case, all coral samples clustered together regardless of disease state and ANOSIM confirmed no significant differences between the healthy and white plague bacterial communities [10]. The larger number of informational OTUs (13,441) that the PhyloChip™ G3 provided was likely important to resolving the health state; however, as shown in Figure 1B, a subset of 313 OTUs containing coral-associated sequences was sufficient to produce a similar outcome.

Many studies, including the two prior studies of coral disease employing PhyloChips™ [25], [26], have shown increased diversity in diseased corals compared to healthy [12], [41], [42]. In this study we found the opposite, with greater numbers of OTUs both present and unique to healthy samples compared to diseased (Table 2). At first we thought that the difference in diversity detected by the PhyloChip™ G3 compared to the PhyloChip™ G2 might be partly due to the presence of the coral-specific OTUs on the G3 chip (Table S1), since they may reveal more microbial community diversity in healthy corals than was possible on the previous chip. Also, it seemed logical that the increased diversity observed with the G3 chip might simply be due to the greater taxonomic coverage: using the G2 PhyloChip, Sunagawa et al. [26] detected 1,702 out of a possible total of 8,741 OTUs versus our G3 data reflecting 13,441 out of a possible 59,222 bacterial OTUs. This is an almost eightfold increase in OTUs and therefore a substantial amount of additional information. However, both those arguments fail when comparing between two G3 studies. In our case, much of the diversity in healthy samples was due to taxa that were represented by a single OTU on the PhyloChip™ G3 (i.e., uncommon families, genera, and species). Further, of the more than 7,000 OTUs unique to healthy samples (Table 2, top panel), most were not shared by more than a few samples (e.g., greater than 4,000 OTUs were only called present in a single coral sample). The Roder et al. study [25] did not consider any OTUs that were not present in more than one sample (analogous to removing singletons from pyrosequencing datasets). For comparative purposes, we recalculated our rankings after removing all singletons (Table 2, bottom panel). While fully half (6,720) of our OTUs were singletons, their removal did not change our observation of higher diversity in healthy samples compared to diseased. It did cause the percentages of shared taxa to increase dramatically (Table 2, bottom panel).

Out of the 13,441 OTUs present across all samples, 106 OTUs were unique to healthy samples and present in more than half of the healthy replicates (Table 3). Of note are the three groups of multiple OTUs within the same genera: 8 for *Micrococcus*, 81 for *Bacillus*, and 9 for *Vibrio*. This matches well with Figure 2 where both families Bacillaceae and Vibrionaceae were represented by a greater proportion of OTUs in healthy samples compared to diseased. A greater abundance (based on relative intensities) of Bacillaceae was also found in healthy corals compared to white plague-like diseased corals by the PhyloChip™ G3 study in the Indo-Pacific [25]. In soil studies, some strains of *Bacillus* have been shown to fix nitrogen, while others may stimulate nitrogen-fixing activities of other bacteria [43], [44]. This might be a functional role for coral-associated *Bacillus* sp. in healthy corals that is disrupted by the transition to a diseased state. Recent studies of white plague-like disease in other coral species give conflicting results in terms of *Vibrios*: a lower number of Vibrionales sequences was seen in diseased *Diploria strigosa* and *Siderastrea siderea* (both Caribbean species) [28], versus a higher abundance of Vibrionaceae in diseased *Pavona duerdeni* and *Porites lutea* (Pacific species) [25]. While *Vibrio* species have been implicated in several coral diseases [29], [45], [46], they have also been shown to be a normal part of the coral microbiome [47]–[50].

There were eight families that occurred in diseased corals but were not present in any of the healthy samples (Table 2): BSV26/SM1H02, Chlamydiaceae, Gallionellaceae, Rarobacteraceae, Scalinduaceae, Solimonaceae, Spirulinaceae, Thermodesulfobiaceae. Unfortunately, in all but one case (Scalinduaceae, 4 OTUs), each family is only represented by a single OTU on the PhyloChip™ G3, making it difficult to assess the significance of these groups. There were only 38 OTUs that were unique to diseased samples and shared by more than half the diseased replicates (Table 4). Included are two cyanobacterial OTUs and one for *Desulfovibrio*, providing some evidence for the ‘atypical black band disease’ that Ainesworth et al. [7] described as having a macroscopic appearance of white plague. Pantos et al. [12] also had noted similarities in 16S rRNA gene clone library sequences between white plague and black band diseases.

There is also noticeable representation of the Rhodobacteraceae family (8 OTUs; Table 4); this family previously had been detected as enriched in white plague samples of *Orbicella faveolata* by Sunagawa et al. [26] using an earlier generation PhyloChip™ microarray and also in white plague-like samples of Indo-Pacific corals by PhyloChip™ G3 [25]. Rhodobacteraceae OTUs were represented by a greater proportion of OTUs in diseased samples compared to healthy (Figure 2). Sequences from the order Rhodobacterales were consistently and significantly enriched in pyrosequencing datasets from white plague diseased *Diploria strigosa* and *Siderastrea siderea* compared to healthy [28] and have also been associated with another white plague-like disease [12]. Further, a meta-analysis of coral-associated sequences [51] found *Rhodobacter* spp. to be associated with several coral diseases, not just white plague. This seems to corroborate the suggestion by Sunagawa et al. [26] that Rhodobacteraceae are opportunists.

In the recent PhyloChip™ G3 comparison of healthy and white plague-like diseased Indo-Pacific corals, several of the bacterial families that showed higher abundance in diseased corals were Clostridiaceae, Corynebacteriaceae, Lachnospiraceae, and Ruminococcaceae [25]. Similarly, we found a higher relative abundance of OTUs from these families in our diseased samples (Figure 2). Families that both appeared in higher relative abundance in our diseased samples (Figure 2) and were also represented by OTUs unique to disease samples (Table 4) were Corynebacteriaceae, Lachnospiraceae, and Streptococcaceae. Unlike Rhodobacteraceae, these families are all Gram-positive, and recent work on white band disease has indicated that the causative pathogen is a Gram-positive microbe [52]. It has been pointed out that it can be difficult to distinguish between white band and white plague in *Acropora* when surrounded by an outbreak of white plague-like disease in other coral species [13]. Further, it has been suggested that given our difficulty in identifying defining causative agents, coral diseases with this phenotype on the Great Barrier Reef should be collectively referred to as white syndromes [53] rather than distinguishing white plague from white band based solely on host identity. In general, the presence and roles of Gram-positive bacterial groups in corals (both healthy and diseased) have been understudied, likely because of a bias towards the recovery of Gram-negative bacteria in many culture media as well as clone libraries. To this point, the focus has been dominated by attention to Gram-negative Proteobacteria. For example, several white plague studies have described a shift in the bacterial community towards Alphaproteobacteria being more heavily represented in diseased corals [12], [26], [28]. We did not observe that in this study, either in terms of higher intensities or in terms of greater numbers of Alphaproteobacterial OTUs. Newer methods such as the PhyloChip™ G3 and pyrosequencing are uncovering the presence and diversity of Gram-positive bacteria in corals, and suggest we may need to look at them more closely.

Two recent coral disease studies [10], [27] have found no significant differences between the bacterial community composition of healthy and diseased corals. In one case, the comparison was based on a total of 67 bacterial OTUs [10], which likely did not provide enough depth of coverage. The large number of shared OTUs revealed by our study (Table 2) could explain this finding, particularly since the community fingerprints used in that study only identified the dominant taxa. The other study used pyrosequencing and so achieved a much greater depth of coverage, but was limited by having only one healthy and one diseased replicate per location [27]. While we expected that our diseased samples might cluster based on geographic location, indicating different pathogens or disease processes, that did not occur. Instead, the diseased samples all cluster together (Figure 1). The clustering is not tight enough to indicate a specific pathogen or even a single group of bacteria, but there is some evidence for a number of bacterial groups affecting the health state (Figure 2). It is also likely that each coral sampled was at a different stage of the disease process, and we currently have no baseline of the changes a disease lesion undergoes as it progresses over time.

*Aurantimonas coralicida* has been described as the causative agent of white plague type II (WPII) [15]. We found no evidence for its association with any of the diseased samples from the Dry Tortugas and only three samples from the Virgin Islands (one diseased, two healthy) were positive for the OTU that includes the *A. coralicida* sequence. Using the second generation (G2) PhyloChip™and clone libraries, Sunagawa et al. [26] were unable to detect *A. coralicida* in either healthy or white plague-affected *Orbicella faveolata*. More recent work using the PhyloChip™ G3 and clone libraries to examine a white plague-like disease in *Pavona duerdeni* and *Porites lutea* also did not find any indication of *A. coralicida*. Similarly, previous work on *O. annularis* using clone libraries failed to detect *A. coralicida* [12]. Deliberate attempts to culture or detect *A. coralicida* using specific PCR primers (on other Caribbean species *Diploria strigosa* and *Siderastrea siderea*) also failed [28]. However, since it was not mentioned in the methods sections of these papers that any effort was made to measure the disease progression rate (the only macroscopic differentiator between WPI, WPII, and WPIII), there is no way to be certain that the diseases being sampled were in fact WPII. It has been suggested that there are likely multiple etiologies that result in these macroscopic symptoms [5], [9], [10], [26], [28], [54]. Alternatively, recently it has been shown that popular fixatives used to preserve tissue after sampling (before DNA extraction) seem biased against *A. coralicida* and other bacteria with a high percentage of GC bases in their DNA, such that they are not detected in a mixed community even when present [55].

## Conclusions

The PhyloChip™ G3 microarray provides a broader taxonomic overview than can be achieved by a traditionally sized clone library and also provides data that can be analyzed without a steep learning curve of bioinformatics, such as is required to handle pyrosequencing datasets. Our results show no evidence for geographically distinct disease states of coral-associated microbiota or for a single causative agent of this white plague-like disease. The higher taxonomic diversity observed in the healthy samples is likely indicative of the natural spatial heterogeneity that has been documented in coral-associated bacterial communities on *O. annularis* [48] as well as other species [25], [56]. The microbial communities of healthy and diseased samples are clearly different, with differential representation of the families Bacillaceae, Corynebacteriaceae, Lachnospiraceae, Rhodobacteraceae, Streptococcaceae, and Vibrionaceae indicating a need to better understand the relationships between coral and Gram-positive bacteria, which appear to have been underappreciated to date.

## Supporting Information

Table S1 PhyloChip™ G3 operational taxonomic units (OTUs) that contain one or more coral-associated sequences.
(XLS)

File S1 OTU sorting program, QueryOTU.pl, written in Perl.
(PL) Click here for additional data file.

File S2 Comma-delineated OTU table provided as input for the QueryOTU.pl program.
(CSV)

File S3 Detailed example for using QueryOTU.pl program to sort PhyloChip™ G3 OTUs.
(DOC)
