# Supplementary material for: Comparing Bacterial Community Composition between Healthy and White Plague-Like Disease States in Orbicella annularis Using PhyloChip™ G3 Microarrays
Source: PLoS One. 2013 Nov 20;8(11):e79801. doi: 10.1371/journal.pone.0079801 (PMC3835879; doi:10.1371/journal.pone.0079801)
Supplement: File S3 — Detailed example for using QueryOTU.pl program to sort PhyloChip™ G3 OTUs. (DOC) [file pone.0079801.s004.doc]

**Supplemental File S4**

**Detailed example for using QueryOTU.pl program to sort PhyloChip™G3 OTUs**

For example, the numbers in Table 2 were determined as follows. In order to determine the total or absolute number of OTUs present across all healthy samples, the QueryOTU.pl script was run with Group A containing all the healthy samples (Table 1) vs. Group B containing the outgroup OTU. The output list was copied back into Excel and then the VLOOKUP function was used to pair the OTU numbers back to the full phylogenetic information contained in the original spreadsheet. This information was then sorted and filtered to find unique phyla, classes, orders, families, genera, and species, and the COUNTA function was used to obtain counts for each. The absolute number of OTUs present in diseased samples was similarly obtained by setting Group A to be all the white plague-like disease samples (Table 1) and Group B to outgroup. To determine OTUs unique to healthy (meaning present in at least one healthy sample but none of the diseased samples) or diseased (present in at least one diseased sample but none of the healthy samples), Group A was all the healthy samples and Group B was all the diseased samples. To determine the shared OTUs (those present in at least one healthy *and* one diseased sample), all 18 samples were placed in Group A and the outgroup sample into Group B, the results copied into Excel, and the SORT and conditional formatting functions were used to manually find and remove OTUs not meeting this criteria.
